# Comparative transcriptome analysis of two rice genotypes differing in their tolerance to saline-alkaline stress

**DOI:** 10.1371/journal.pone.0243112

**Published:** 2020-12-01

**Authors:** Qian Li, Changkun Ma, Huanhuan Tai, Huan Qiu, An Yang

**Affiliations:** 1 College of Horticulture, Northwest A&F University, Yangling, Shaanxi, China; 2 State Key Laboratory of Eco-hydraulic Engineering in Arid Area, Xi’an University of Technology, Xi’an, China; 3 College of Agronomy, Northwest A&F University, Yangling, Shaanxi, China; 4 State Key Laboratory of Vegetation and Environmental Change, Institute of Botany, the Chinese Academy of Sciences, Beijing, China; Louisiana State University College of Agriculture, UNITED STATES

## Abstract

Saline-alkaline stress is an abiotic stress that suppresses rice plant growth and reduces yield. However, few studies have investigated the mechanism by which rice plants respond to saline-alkaline stress at a global transcriptional level. Dongdao-4 and Jigeng-88, which differ in their tolerance to saline-alkaline stress, were used to explore gene expression differences under saline-alkaline stress by RNA-seq technology. In seedlings of Dongdao-4 and Jigeng-88, 3523 and 4066 genes with differential levels of expression were detected, respectively. A total of 799 genes were upregulated in the shoots of both Dongdao-4 and Jigeng-88, while 411 genes were upregulated in the roots of both genotypes. Among the downregulated genes in Dongdao-4 and Jigeng-88, a total of 453 and 372 genes were found in shoots and roots, respectively. Gene ontology (GO) analysis showed that upregulated genes were enriched in several GO terms such as response to stress, response to jasmonic acid, organic acid metabolic process, nicotianamine biosynthetic process, and iron homeostasis. The downregulated genes were enriched in several GO terms, such as photosynthesis and response to reactive oxygen species. Kyoto Encyclopedia of Genes and Genomes (KEGG) pathway analysis revealed that Dongdao-4 seedlings were specifically enriched in the biosynthesis of secondary metabolites such as diterpenoids and phenylpropanoids. The upregulated genes that were involved in secondary metabolite biosynthesis, amino acid biosynthesis, betalain biosynthesis, organic acid metabolic process, and iron homeostasis pathways may be central to saline-alkaline tolerance in both rice genotypes. In contrast, the genes involved in the diterpenoid and phenylpropanoid biosynthesis pathways may contribute to the greater tolerance to saline-alkaline stress in Dongdao-4 seedlings than in Jigeng-88. These results suggest that Dongdao-4 was equipped with a more efficient mechanism involved in multiple biological processes to adapt to saline-alkaline stress.

## Introduction

Soil salinization-alkalization threatens agricultural production worldwide, causing considerable damage to crop growth and loss of crop yield [1, 2]. Based on data from the Food and Agriculture Organization/UNESCO, 831 million ha of soils globally are affected by saline-alkaline stress [3]. Due to climate change and unsustainable cultivation practices, increased salinization of arable land is expected which could result in 50% land loss by the year 2050 [4]. Rice is more sensitive to saline-alkaline stress than barley and wheat, especially in the seedling and reproductive stages [5]. Therefore, elucidation of the physiological and molecular mechanisms of the rice plant response and adaptation to saline-alkaline stress will provide important clues for the selection and breeding of crop genotypes capable of growth in saline-alkaline soils.

To adapt to salt stress, plants are equipped with several mechanisms such as compartmentation of Na^+^ into vacuoles, increased activities of antioxidant enzymes and accumulation of compatible solutes (e.g., proline and soluble sugars) [6, 7]. Transcriptome changes related to these physiological processes under salt stress have been reported [8–10]. Under alkaline conditions, plants are exposed to high soil pH, thereby suffering from iron (Fe) deficiency. Fe deficiency responsive genes are reported to be involved in plant adaptation to alkaline conditions [11, 12]. Saline-alkaline stress is more complex than individual salt and alkaline stresses due to the presence of high levels of toxic Na^+^, osmotic stress, and high soil pH [1]. To adapt to saline-alkaline stress, plants have evolved numerous strategies to cope with ionic stress, osmotic stress, oxidative stress, and high soil pH. Under saline-alkaline stress conditions, maintaining cellular ion homeostasis is an important mechanism that confers tolerance in plants. Several studies have revealed that maintaining a low Na^+^ concentration in shoots contributes to the greater tolerance of saline-alkaline stress in plants [13, 14]. Osmotic homeostasis and reactive oxygen species (ROS) scavenging have been reported to play important roles in saline-alkaline stress tolerance. Li et al. (2020) [15] reported that overexpression of *VaERF3* induces saline-alkaline tolerance in transgenic Arabidopsis by enhancing the accumulation of proline and the activity of antioxidant enzymes. In saline-alkaline soil, plants have not only evolved mechanisms to adapt to ionic stress, osmotic stress, and oxidative stress, but have also evolved strategies to cope with nutrient stress due to the high soil pH that results from the hydrolysis of NaHCO_3_ and Na_2_CO_3_. Iron acquisition, nitrate assimilation, and calcium metabolism have been reported to be critical mechanisms in tolerance to saline-alkaline stress [16–18]. Moreover, the accumulation of organic acids (such as citric acid) and secretion of H^+^ was higher in saline-alkaline-tolerant plants than in saline-alkaline-sensitive plants [16, 19]. In addition, the integrity of chloroplast structure and the activation of photosynthesis have been reported as crucial for saline-alkaline resistance [20].

RNA-Seq technology is a powerful tool for revealing gene networks associated with the response of plants to stress and numerous genes have been shown to be regulated by saline-alkaline stress in alfalfa [21, 22], alkaligrass [23], and peach [20]. However, there are few reports that have evaluated the effect of saline-alkaline stress in rice at a global transcriptional level. In our previous studies, we evaluated the effect of saline-alkaline stress on the growth and germination in rice genotypes. We found that Dongdao-4 is a saline-alkaline tolerant stress genotype due to the greater efficiency in acquiring iron under saline-alkaline conditions at the seeding stage, while Jigeng-88 is a relatively saline-alkaline-sensitive genotype [16, 24, 25]. In this study, these two rice genotypes differing in their tolerance to saline-alkaline stress were compared at the global transcriptional level by RNA-Seq.

## Materials and methods

### Plant growth and treatment

Dongdao-4 and Jigeng-88 seeds were germinated in tap water at 37°C for 2 days in the dark and placed on moist tissue paper for 2 days at 30°C. The germinated seedlings were then transferred to nutrient solution containing 1.425 mM NH_4_NO_3_, 0.42 mM NaH_2_PO_4_, 0.510 mM K_2_SO_4_, 0.998 mM CaCl_2_, 1.643 mM MgSO_4_, 0.168 mM Na_2_SiO_3_, 0.125 mM Fe-EDTA, 0.019 mM H_3_BO_3_, 0.009 mM MnCl_2_, 0.155 mM CuSO_4_, 0.152 mM ZnSO_4_, and 0.075 mM Na_2_MoO_4_. The seedlings were then placed in a growth chamber maintained at 30°C/22°C (day/night) with a 14-h photoperiod and relative humidity maintained at approximately 70%.

To compare the effect of saline-alkaline stress on rice seedlings at the global transcriptional level, Dongdao-4 and Jigeng-88 seedlings were grown in normal culture solution for 3 weeks. Half of the seedlings were transferred to culture solution supplemented with 40 mM Na^+^ (10 mM Na_2_CO_3_ and 20 mM NaCl) for 1 day and then exposed to 60 mM Na^+^ (10 mM Na_2_CO_3_ and 40 mM NaCl) for 1 week. The remaining seedlings kept in the normal culture solution were used as control plants. The solution was refreshed daily. The pH of the control hydroponic solution was adjusted to 6.0, while the solution used for saline-alkaline treatment was adjusted to pH 8.5.

### RNA isolation, cDNA library construct and Illumina deep sequencing

The methods used for RNA isolation are described in Li et al. (2016) [16]. Two biological replicates were used for each genotype. The total RNA of shoots and roots was extracted from Dongdao-4 and Jigeng-88 seedlings using RNAiso reagent (TaKaRa Biotechnology Co., Ltd., Kyoto, Japan). RNA integrity and quality were assayed with agarose gel electrophoresis and a Bioanalyzer using an Agilent RNA 6000 Nano Chip (Agilent Technologies, Santa Clara, CA, USA). Samples with RNA integrity number values >8 were used for reverse- transcription into first-strand cDNA. cDNA libraries were prepared according to the manufacturer’s protocol (Illumina, San Diego, CA, USA) and assayed using the BioAnalyzer 2100 system and qPCR (Kapa Biosystems, Woburn, MA, USA). Subsequently, the libraries were sequenced on an Illumina HiSeq 2000 instrument.

### Sequencing data analysis

Raw reads were filtered to remove adaptors, tags of reads and low-quality reads. Then, the remaining high-quality reads were used for mapping analysis against the reference genome sequences (ftp://ftp.ensemblgenomes.org/pub/plants/release-30, last accessed November 30, 2015) using Tophat [26]. The empirical criterion of a greater than two fold change and a significant q value (false discovery rate-adjusted P value) of <0.05 based on two independent biological replicates were used to estimate differentially expressed genes (DEGs). GO enrichment was performed using Cytoscape software (http://www.cytoscape.org, version 2.5.2) with the Bingo plugin (http://www.psb.ugent.be/cbd/papers/BiNGO/, version 2.3). Hypergeometric tests with a Benjamini Hochberg false discovery rate (FDR) were performed using the default parameters to adjust the P-value. KEGG enrichment was performed using BLAST comparisons against the KEGG GENES database KAAS (KEGG Automatic Annotation Server). RNA-seq data have been deposited in the NCBI sequencing read archive (https://www.ncbi.nlm.nih.gov/bioproject/PRJNA665812; Accession: PRJNA665812).

### Real-time RT-PCR

The total RNA of shoots and roots was extracted from Dongdao-4 and Jigeng-88 seedlings using RNAiso reagent and reverse-transcribed into first-strand cDNA with a PrimeScript^®^ RT Reagent kit (TaKaRa Biotechnology Co., Ltd., Kyoto, Japan). Three biological replicates were used for each genotype. Real-time RT-PCR was performed following the protocols described in Li et al. (2016) [16]. All the primers used for quantitative RT-PCR are listed in S1 Table in S1 File. The relative expression level was analyzed by the comparative Ct method.

## Results

### RNA-seq analysis

To gain a better understanding of the mechanism underlying tolerance to saline-alkaline stress at a global transcriptional level, transcriptomic changes in seedlings of Dongdao-4 and Jigeng-88 were investigated by RNA-Seq. A total of 905 million reads were generated. More than 869 million clean reads were retained for assembly and further analysis. Among all the reads, more than 86% had Phred-like quality scores at the Q30 level (an error probability of 0.1%) (Table 1). The sequencing reads were quality checked, and ~fifty million of the remaining high-quality reads in each transcriptome sample were mapped to unique positions on the rice reference genome (Table 1).

**Table 1:** Summary of sequencing results and their matches in the *Oryza sativa japonica*.

Treatment	Sample name	Clean reads	Q30%	Total mapped reads	Clean reads rate (%)
Control	Dongdao-4-Shoot-1	54,137,962	92.20	50,926,791	97.99
	Dongdao-4-Shoot-2	57,208,312	92.66	53,987,360	98.09
	Jigeng-88-Shoot-1	56,784,834	89.69	53,001,156	97.15
	Jigeng-88-Shoot-2	53,432,852	90.41	49,824,285	97.32
	Dongdao-4-Root-1	57,462,090	90.72	53,283,879	96.04
	Dongdao-4-Root-2	53,831,806	91.00	49,951,648	94.7
	Jigeng-88-Root-1	51,506,256	87.85	46,736,878	92.94
	Jigeng-88-Root-2	56,149,974	90.31	52,408,232	95.99
Saline-alkaline stress	Dongdao-4-Shoot-1	54,774,108	90.96	51,226,121	96.76
	Dongdao-4-Shoot-2	52,910,916	90.50	49,579,340	95.59
	Jigeng-88-Shoot-1	53,520,734	90.47	50,162,643	95.04
	Jigeng-88-Shoot-2	53,756,234	90.28	50,389,955	96.3
	Dongdao-4-Root-1	56,291,970	89.89	51,984,770	97.17
	Dongdao-4-Root-2	55,396,538	89.56	51,107,666	95.06
	Jigeng-88-Root-1	52,904,614	86.27	48,017,802	95.46
	Jigeng-88-Root-2	49,199,682	88.91	45,409,151	94.04
Average		54,329,305	90.11	50,499,855	95.98

‘Nipponbare’ genome.

**Fig 1:**
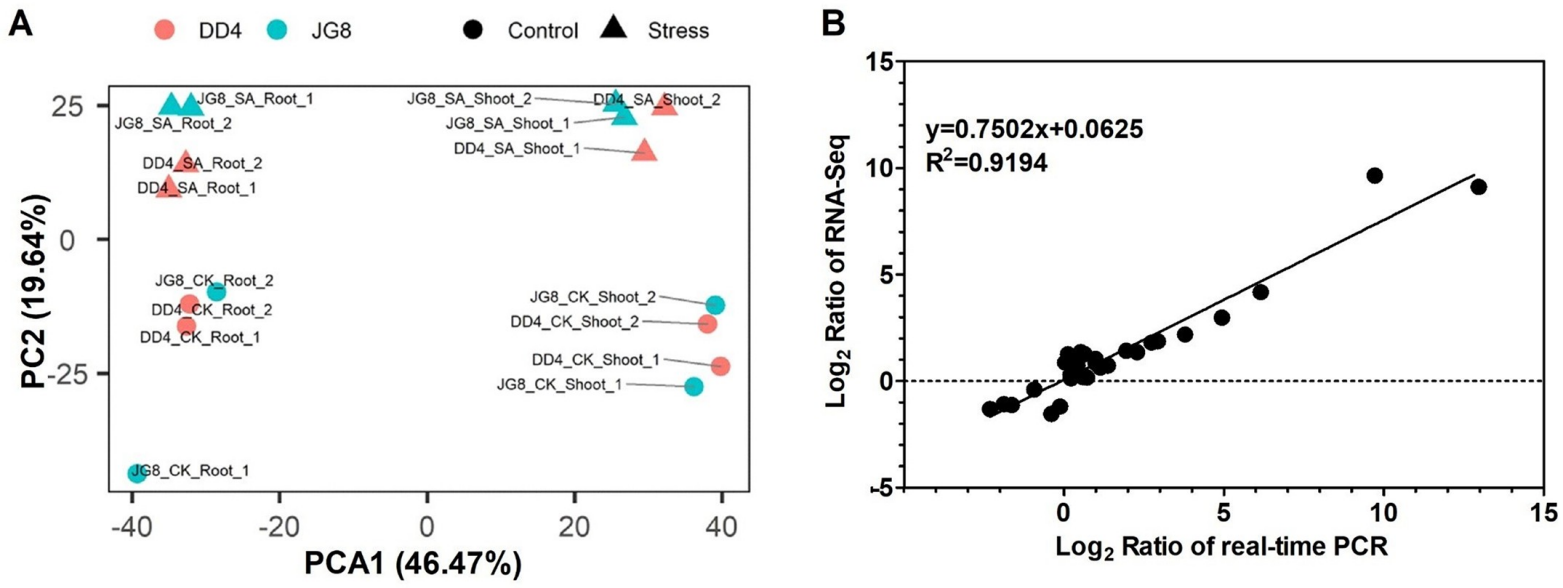
Verification of RNA-Seq results through (A) principal component analysis and (B) real-time PCR. Correlation between data obtained from RNA-Seq and RT-PCR data. Data are the mean of three biological replicates.

Furthermore, principal component analysis (PCA) was used to evaluate the relationships among transcriptome samples. As shown in Fig 1A, the results revealed that the relationships among the two biological replicates of each genotype combination were close. In addition, real-time PCR analyses were performed to validate the data from RNA-sequencing. We randomly selected 19 DEGs to verify RNA-sequencing data by real-time PCR analysis. The ratios of stress to control are presented in Fig 1B. Pearson correlation analysis showed that the results generated by the two methods were highly correlated (Pearson correlation coefficients R^2^ = 0.92; Fig 1B), which suggested that the RNA-sequencing data were reliable and reproducible. Both analyses indicated that the throughput and sequencing quality were appropriate for further analysis.

### Identification of DEGs in response to saline-alkaline stress

**Fig 2:**
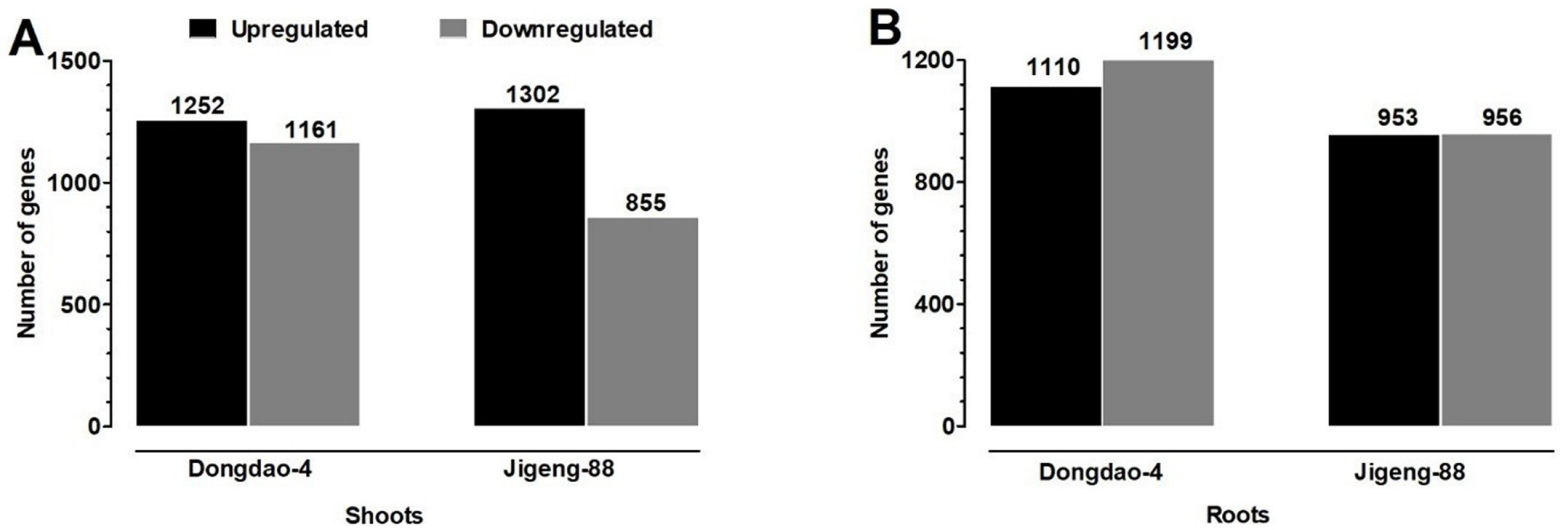
Summary of the numbers of differentially expressed genes upon exposure to saline-alkaline stress in Dongdao-4 and Jigeng-88. (A) The number of genes up- or downregulated in shoots of Dongdao-4 and Jigeng-88. (B) The number of genes up- or downregulated in roots of Dongdao-4 and Jigeng-88.

Genes were deemed to be DEGs if their transcript levels were 2-fold greater (*P* < 0.05 in Student’s *t*-tests) under saline-alkaline stress than under control conditions. According to this criterion, there were 2413 DEGs in the shoot of Dongdao-4, of which 1252 were upregulated and 1161 were downregulated (Fig 2A). In the shoot of Jigeng-88 seedlings, 2157 DEGs were detected, with 1302 genes upregulated and 855 downregulated genes (Fig 2A). In the case of the Dongdao-4 root, there were 1110 upregulated and 1199 downregulated genes (Fig 2B). In the root of Jigeng-88 seedlings, 953 were upregulated and 956 genes were downregulated (Fig 2B).

**Fig 3:**
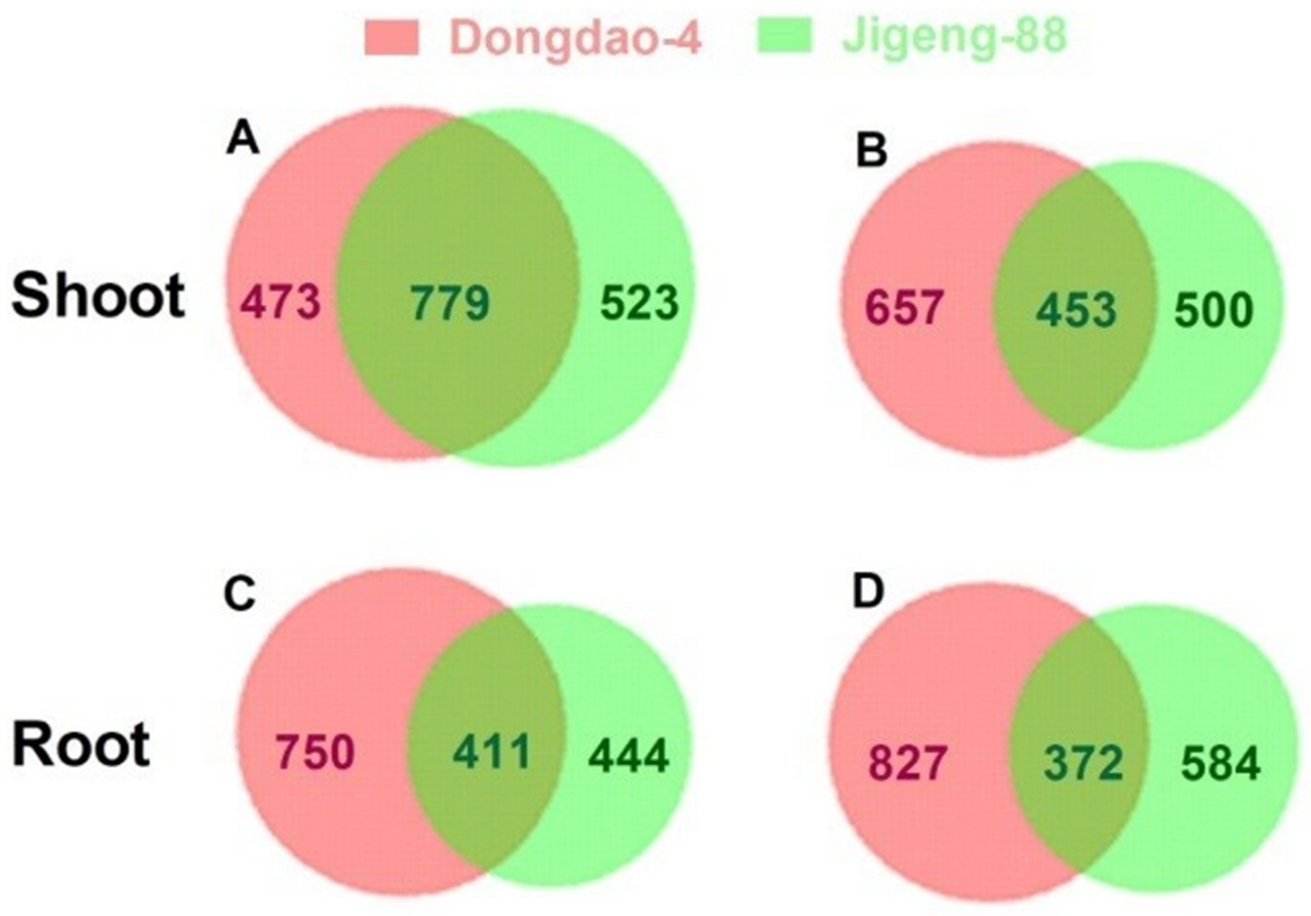
Venn diagram of differentially expressed genes (DEGs) in Dongdao-4 and Jigeng-88. (A) Upregulated DEGs in shoots; (B) downregulated DEGs in shoot; (C) upregulated DEGs in roots; (D) downregulated DEGs in roots. The numbers of genes shared and distinct to each genotype are shown.

A total of 473 and 523 genes were specifically upregulated in the shoots of Dongdao-4 and Jigeng-88 seedlings, respectively (Fig 3A, S2 and S3 Tables in S1 File). There were 799 genes that were induced in shoots of both rice genotypes, with 142 of these genes showing greater transcript levels in Dongdao-4 than in Jigeng-88 (Fig 3A, S4 Table in S1 File). In addition, the transcript levels of 657 and 500 genes were specifically reduced in the shoots of Dongdao-4 and Jigeng-88 seedlings, respectively (Fig 3B, S5 and S6 Tables in S1 File). A total of 453 genes were downregulated in the shoots of both rice genotypes, with 121 of them having greater transcript levels in Jigeng-88 than Dongdao-4 (Fig 3B, S7 Table in S1 File). In the roots of Dongdao-4 and Jigeng-88, 750 and 444 genes were specifically upregulated, respectively (Fig 3C, S8 and S9 Tables in S1 File). There were 411 genes that were induced in the shoots of both rice genotypes, with 97 genes having higher transcriptional levels in Dongdao-4 than Jigeng-88 (Fig 3C, S10 Table in S1 File). Of the downregulated genes in the roots of Dongdao-4 and in Jigeng-88 seedlings, the transcription levels of 827 and 584 genes were specifically reduced, respectively (Fig 3D, S11 and S12 Tables in S1 File). A total of 372 genes were downregulated in the roots of both rice genotypes, with 83 of these genes having greater transcriptional levels in Jigeng-88 than Dongdao-4 (Fig 3D, S13 Table in S1 File).

### Gene ontology (GO) categorization of DEGs

Gene Ontology (GO) analysis was conducted to obtain an overview of the functional information of DEGs. For the common upregulated DEGs, 5 and 6 GO terms associated with biological process were significantly enriched in shoots and roots, respectively (Table 2). The higher number of enriched terms in the roots than in the shoots indicated that the roots were more sensitive to saline-alkaline stress. There were 5 GO terms in shoots which were involved in response to stress, response to jasmonic acid and molecule catabolic process (Table 2). In contrast, the 8 GO terms in roots were involved in metabolic processes such as organonitrogen compound metabolic processes, organic acid metabolic processes, the nicotianamine biosynthetic process, iron homeostasis, etc. (Table 2). Among the downregulated genes in shoots and roots, there were 3 and 2 GO terms, respectively, that were significantly enriched in biological processes (Table 3). The 3 GO terms in shoots were involved in photosynthesis, while the 2 GO terms enriched in roots were involved in the response to reactive oxygen species.

**Table 2:** Functional category annotations of upregulated genes in both rice genotypes.

Go categories	Shoot				Root			
	GO terms	DEGs in each GO term	Genes in background	FDR	GO terms	DEGs in each GO term	Genes in background	FDR
Biological process	response to stress	129	3738	0.002	organonitrogen compound metabolic processes	44	1540	0.003
	response to jasmonic acid	18	195	0.002	organic acid metabolic processes	44	1552	0.003
	defense response	68	1805	0.032	nicotianamine biosynthetic process	3	3	0.003
	organonitrogen compound catabolic process	22	327	0.012	tricarboxylic acid biosynthetic process	3	3	0.003
	cell wall macromolecule catabolic process	6	25	0.024	oxoacid metabolic process	42	1541	0.005
					amino acid salvage	4	10	0.005
					L-methionine biosynthetic process	4	10	0.012
					iron homeostasis	13	48	0.021
Molecular function	chitin binding	7.00	32.00	0.03				

Note: FDR = false discovery rate.

**Table 3:** Functional category annotations of downregulated genes in both rice genotypes.

Go categories	Shoot				Root			
	GO terms	DEGs in each GO term	Genes in background	FDR	GO terms	DEGs in each GO term	Genes in background	FDR
Biological process	photosynthesis	25	261	0.000	response to ROS	15	321	0.002
	protein-chromophore linkage	11	66	0.000	ROS metabolic process	12	266	0.015
	generation of precursor metabolites and energy	18	368	0.001				
Cellular component	photosynthetic membrane	32	312	0.000				
	photosystem I	10	32	0.000				
	photosystem II	16	80	0.000				
	chloroplast stroma	23	488	0.000				
	plastid stroma	23	493	0.000				
	organelle subcompartment	36	455	0.000				
	nucleosome	8	88	0.001				
	DNA packaging complex	8	92	0.001				
	macromolecular complex	56	2888	0.020				
	extracellular region	45	2018	0.004	extracellular region	42	2018	0.017
	apoplast	16	451	0.007				
	cell wall	21	782	0.029				
	external encapsulating structure	21	788	0.031				
Molecular function	chlorophyll binding	10	35	0.000	antioxidant activity	13	257	0.002
					tetrapyrrole binding	21	781	0.018

Note: FDR = false discovery rate.

**Fig 4:**
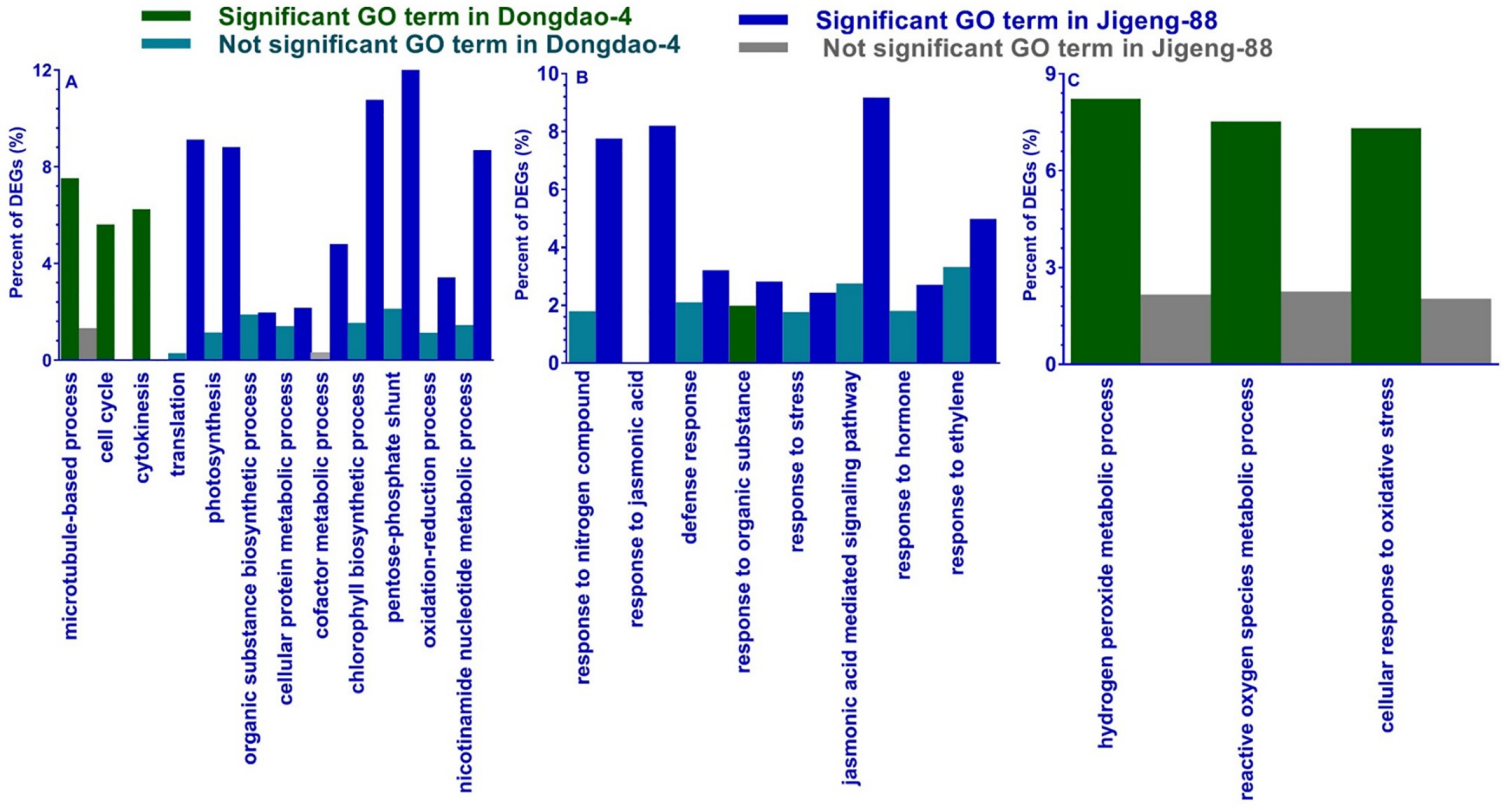
Significantly enriched GO terms at the biological process ontology level for differentially regulated genes in Dongdao-4 and Jigeng-88. (A) GO terms specifically enriched by downregulated DEGs in shoots, (B) GO terms specifically enriched by upregulated DEGs in roots, (C) GO terms specifically downregulated DEGs in roots. GO terms were defined as significantly enriched if the false discovery rate (FDR) was ≤ 0.05. Green and red bars represent significantly enriched GO terms in Dongdao-4 and Jigeng-88, respectively. Blue and gray bars represent GO terms that are not significantly enriched in Dongdao-4 and Jigeng-88, respectively.

We also conducted GO analysis of specifically upregulated genes in the shoots of Dongdao-4 and Jigeng-88 seedlings, and found that no GO terms were enriched. Among the specifically downregulated genes, a total of 3 and 9 GO terms related to biological processes were significantly enriched in the shoots of Dongdao-4 and Jigeng-88 seedlings, respectively (Fig 4A). The downregulated genes in the shoots of Dongdao-4 were involved in the cell cycle and cytokinesis, and those in Jigeng-88 were involved in photosynthesis, the pentose-phosphate shunt, and translation (Fig 4A). In the roots of Dongdao-4 seedlings, one GO term (response to organic substance) was enriched among the specifically upregulated genes. For the 500 specifically upregulated DEGs in roots of Jigeng-88 seedlings, 8 GO terms related to biological process were significantly enriched and were involved in response to stress and hormones (Fig 4B). For the 827 specifically downregulated genes in the roots of Dongdao-4 seedlings, there were 3 GO terms that were all involved in the response to reactive oxygen species (Fig 4C). There were no enriched GO terms among the specifically downregulated genes in Jigeng-88 roots.

### Kyoto Encyclopedia of Genes and Genomes (KEGG) pathway annotation

**Fig 5:**
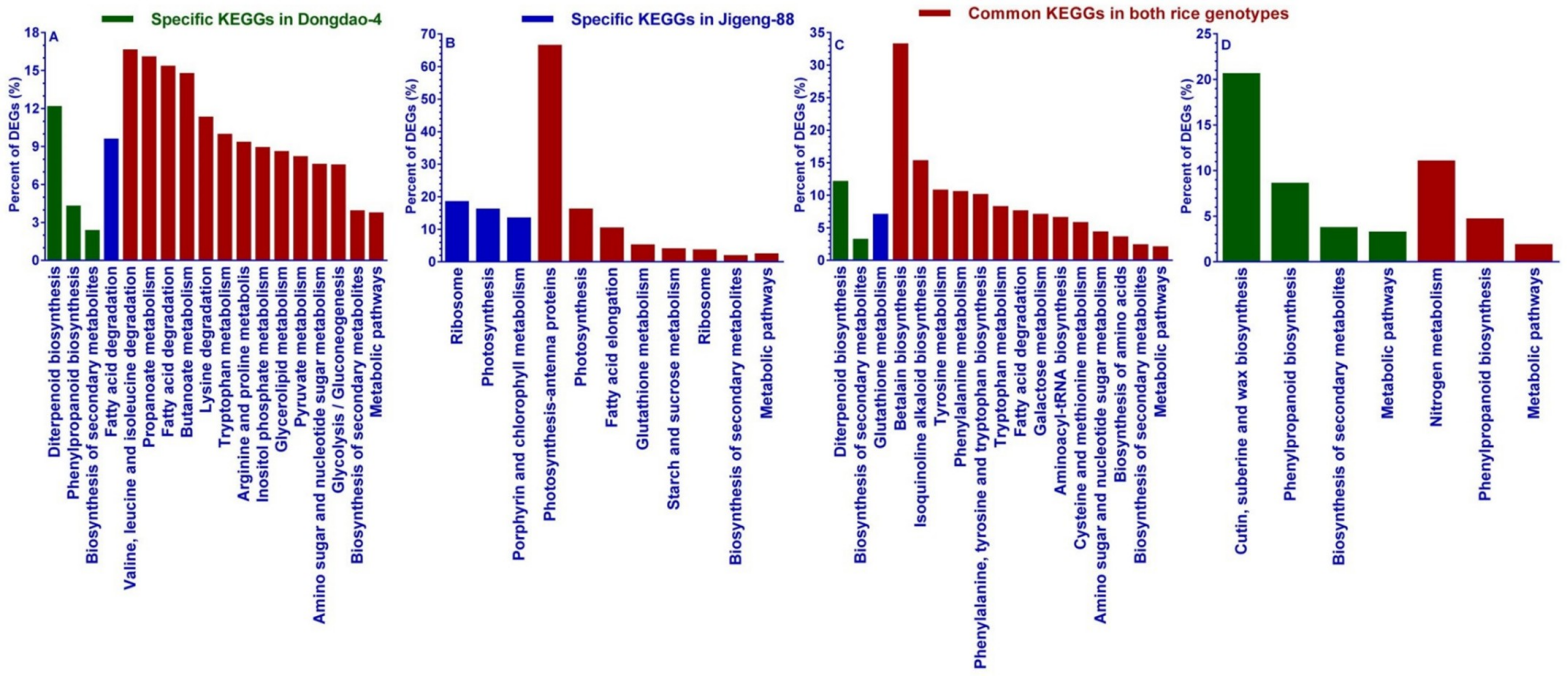
KEGG terms significantly enriched by differentially regulated genes in Dongdao-4 and Jigeng-88. (A) KEGG terms specifically enriched by upregulated DEGs in shoots, (B) KEGG terms specifically enriched by downregulated DEGs in shoots, (C) KEGG terms specifically enriched by upregulated DEGs in roots, (D) KEGG terms specifically enriched by downregulated DEGs in roots.

We carried out a Kyoto Encyclopedia of Genes and Genomes (KEGG) pathway analysis to uncover the signaling pathways involved in saline-alkaline stress in Dongdao-4 and Jigeng-88 seedlings. As shown in Fig 5A, 473 upregulated genes specifically in Dongdao-4 shoots were significantly enriched in 3 KEGG terms, i.e., diterpenoid biosynthesis, biosynthesis of secondary metabolites, and phenylpropanoid biosynthesis (Fig 5A). In contrast, all 523 upregulated genes specifically only in the shoots of Jigeng-88 seedlings were significantly enriched in only one KEGG term, i.e., fatty acid degradation (Fig 5A). Among the 799 upregulated genes in the shoots of Dongdao-4 and Jigeng-88, 14 KEGG terms such as valine, leucine and isoleucine degradation, propanoate metabolism, and fatty acid degradation were significantly enriched (Fig 5A). No KEGG term was significantly enriched for 657 specific downregulated genes in Dongdao-4 shoot. However, the 500 genes specific downregulated in Jigeng-88 shoot were significantly enriched in 3 KEGG terms, namely ribosomes, photosynthesis and porphyrin, and chlorophyll metabolism (Fig 5B). For the 453 common downregulated genes in shoots of both genotypes, 8 KEGG terms were significantly enriched with photosynthesis-antenna proteins, photosynthesis, and fatty acid elongation being the most enriched KEGG terms. (Fig 5B). The 750 root-specific upregulated genes of Dongdao-4 were assigned to 2 KEGG terms, i.e., genes related to biosynthesis of secondary metabolites and diterpenoid biosynthesis (Fig 5C), and the 444 root-specific upregulated genes of Jigeng-88 were significantly enriched in only one KEGG term, namely glutathione metabolism (Fig 5C). For the 411 common upregulated genes in roots of Dongdao-4 and Jigeng-88 seedlings, 14 KEGG terms were significantly enriched, including betalain biosynthesis, secondary metabolites, and amino acids biosynthesis (Fig 5C). For the 827 specific downregulated genes in roots of Dongdao-4 seedlings, 4 KEGG terms were significantly enriched for phenylpropanoid biosynthesis, cutin, suberin and wax biosynthesis, metabolic pathways, and biosynthesis of secondary metabolites (Fig 5D). However, no KEGG terms were significantly enriched for the 584 specifically downregulated genes in root of Jigeng-88 seedlings (Fig 5D). The 372 downregulated genes in roots of both genotypes were significantly enriched in 3 KEGG terms, i.e., genes related to metabolic pathways, phenylpropanoid biosynthesis, and nitrogen metabolism (Fig 5D).

### Differential expression of genes involved in iron homeostasis

Maintaining iron homeostasis is essential for rice growth under saline-alkaline conditions. Among upregulated genes in both rice genotypes, the functional category ‘nicotianamine biosynthetic process’ was enriched. Nicotianamine biosynthesis is critical for phytosiderophore mediated iron acquisition. We detected genes involved in the nicotianamine biosynthetic process and other processes related to iron acquisition and translocation. A total of 12 and 18 DEGs involved in iron acquisition and translocation were found in shoot and root of Dongdao-4, respectively (Table 4). In shoot and root of Jigeng-88, 9 and 18 DEGs involved in iron acquisition and translocation were identified, respectively (Table 4). In shoots of both rice genotypes, genes involved in iron homeostasis, including *OsYSL2*, *OsYSL15*, *OsIRO2*, *OsNAAT3*, *OsNAS1*, and *OsNAS2*, were significantly increased, particularly in Dongdao-4 compared to Jigeng-88 (Table 4). Genes involved in iron acquisition and translocation, such as *OsIRT1*, *OsIRT2*, *OsYSL2*, *OsYSL15*, *OsIRO2*, *OsNAAT3*, *OsNAS1*, and *OsNAS2*, were significantly increased in the roots of both genotypes, but the expression was higher in Dongdao-4 than in Jigeng-88 (Table 4).

**Table 4:** Differentially regulated genes involved in iron homeostasis in Dongdao-4 and Jigeng-88 seedlings.

Gene ID	Annotation	Shoots				Roots			
		Dongdao-4		Jigeng-88		Dongdao-4		Jigeng-88	
		log_2_FC	FDR	log_2_FC	FDR	log_2_FC	FDR	log_2_FC	FDR
Os01g0952800	OsIRO2	11.13	0.000	7.16	0.000	6.94	0.000	4.60	0.000
Os03g0379300	OsIRO3	-0.65	0.085	1.51	0.000	2.23	0.000	3.73	0.000
Os02g0650300	OsYSL15	10.91	0.009	5.55	0.007	14.40	0.000	6.16	0.001
Os03g0667500	OsIRT1					9.11	0.000	3.64	0.000
Os03g0667300	OsIRT2		0.035		0.059	10.89	0.000	6.88	0.000
Os02g0649900	OsYSL2	3.09	0.000	0.87	0.625	13.85	0.000	2.06	0.504
Os02g0302200	OsNAAT3	1.20	0.000	0.49	0.473	1.63	0.000	1.94	0.028
Os03g0307300	OsNAS1	19.69	0.000	6.29	0.000	14.26	0.000	6.38	0.000
Os03g0307200	OsNAS2	103.16	0.000	8.98	0.000	14.11	0.000	5.38	0.000
Os07g0689600	OsNAS3	0.69	0.011	0.92	0.061	3.23	0.000	2.62	0.000
Os07g0258400	OsNRAMP1	2.66	0.000	7.69	0.000	7.39	0.000	4.32	0.000
Os03g0208500	OsNRAMP2					1.73	0.000	1.40	0.001
Os01g0733001	OsNRAMP3	1.08	0.016	0.36	0.853	-1.44	0.002	-1.36	0.036
Os01g0503400	OsNRAMP4					1.29	0.000	1.30	0.006
Os07g0257200	OsNRAMP5	-1.32	0.040	-2.14	0.000				
Os09g0396900	OsVIT2	-1.79	0.000	-2.27	0.000	-5.03	0.000	-5.31	0.000
Os04g0686800		-1.26	0.030	-0.48	0.861	-1.53	0.000	-2.58	0.003
Os07g0467200		-0.66	0.056	-2.11	0.000	-0.42	0.647	-2.05	0.013
Os07g0628500						-1.88	0.000	-0.80	0.638
Os04g0538400						-7.03	0.000	-7.68	0.000
Os11g0601700						-0.95	0.000	-1.39	0.000

Note: log_2_FC = log_2_ Fold Change, FDR = false discovery rate.

### Differential expression of genes involved in Gibberellin (GA) and phenylpropanoid biosynthesis

GA is known as an important hormone in plants during responses to abiotic stresses such as cold stress, salt stress, and drought stress. Among the genes specifically upregulated in Dongdao-4, one KEGG term, diterpenoid biosynthesis, which is involved in GA biosynthesis, was significantly enriched. A gene involved in GA biosynthesis (*Os03g0856700*) was upregulated by saline-alkaline stress (Table 5). In addition, among the genes specifically upregulated in Dongdao-4, genes involved in phenylpropanoid metabolism were also upregulated by saline-alkaline stress (Table 5).

**Table 5:** The upregulated genes involved in GA and phenylpropanoid biosynthesis in Dongdao-4 seedlings.

Gene ID	Annotation	Shoots		Roots	
		log_2_FC	FDR	log_2_FC	FDR
Os12g0491800	Ent-sandaracopimara-8(14),15-diene synthase	1.90	0.000		0.000
Os04g0611700	Ent-kaurene synthase-like 3	2.88	0.000		
Os06g0570100	Ent-kaurene oxidase 2	1.44	0.000		
Os02g0571300	Ent-pimara-8(14),15-diene synthase	2.08	0.000		
Os03g0856700	Gibberellin 20 oxidase 1	1.37	0.000		
Os02g0570900	Ent-copalyl diphosphate synthase 2				0.042
Os06g0569500	Ent-sandaracopimaradiene 3-hydroxylase			2.19	0.000
Os02g0570400	Ent-cassa-12,15-diene synthase			3.72	0.005
Os06g0110000	Ent-kaurenoic acid oxidase 1			1.47	0.000
Os04g0474800	Beta-glucosidase 12	1.93	0.000		
Os01g0283700	Cinnamoyl-CoA reductase 1	1.68	0.000		
Os08g0509200	Beta-glucosidase 27	1.67	0.000		
Os09g0399800	Probable cinnamyl alcohol dehydrogenase 8A	5.23	0.000		
Os07g0677400	Peroxidase 2	2.44	0.001		
Os04g0175600	Probable inactive methyltransferase	1.06	0.011		
Os01g0930800	Beta-glucosidase 5	1.16	0.002		
Os08g0270400	Putative cinnamyl alcohol dehydrogenase 5	3.69	0.000		
Os07g0677200	Peroxidase 2	1.76	0.000		
Os04g0229100	Probable cinnamyl alcohol dehydrogenase 6	1.79	0.000		

Note: log_2_FC = log_2_ Fold Change, FDR = false discovery rate.

## Discussion

In recent years, numerous studies have been conducted to elucidate the mechanisms by which plants adapt to saline-alkaline stress. However, there have been few reports that have evaluated the effect of saline-alkaline stress on rice seedlings at the global transcriptional level. We reported in our earlier study that an efficient Fe acquisition system was involved in tolerance to saline-alkaline stress in Dongdao-4 plants [16, 24, 25]. In the present study, the mechanisms underlying this greater saline-alkaline tolerance in Dongdao-4 were investigated at the global transcriptional level. Among these DEGs, genes involved in the response to stress, response to jasmonic acid, organic acid metabolic, iron homeostasis and betalain biosynthesis were upregulated in both Dongdao-4 and Jigeng-88. These results indicated that these common upregulated genes may be implicated in tolerance to saline-alkaline stress in both rice genotypes (Tables 2 and 4 and Fig 5). In addition, we determined that numerous regulated genes in Dongdao-4 were involved in GA and phenylpropanoid biosynthesis (Fig 5 and Table 5). The genes involved in diterpenoid and phenylpropanoid biosynthesis may underlie the greater tolerance of Dongdao-4 seedlings to saline-alkaline stress.

Among the 799 common upregulated genes in shoots of both genotypes, 142 genes had magnitudes of induction that were higher in Dongdao-4 than in Jigeng-88. Among these genes, we found several transcription factors such as MYB, WRKY and MADs-box transcription factors that are involved in abiotic stress. For example, *Os04g0304400* encodes OsMADS25 and imparts salinity tolerance in rice through elevated activity of antioxidant enzymes, increased accumulation of the osmoprotective solute proline, and reductions in the number of open stomata [27]. OsWRKY8, which is encoded by *Os05g0583000*, is involved in osmotic stress tolerance. The overexpression of *OsWRKY8* increased tolerance to osmotic stress in Arabidopsis by modulating ABA-independent responsive genes [28]. *Os11g0684000* encodes OsJAMyb and is a JA-inducible gene. Yokotani et al. (2013) [29] reported that OsJAMyb not only functioned in resistance to blast infection but was also involved in osmotic adjustment, ROS removal, and ion homeostasis. In addition to transcription factors, other abiotic stress-responsive genes were observed, such as *Os12g0150200*, *Os12g0569700*, and *Os02g0644000*. *Os12g0150200* encodes CYP94C2b and belongs to the *CytP450* gene family. CYP94C2b delays stress-induced leaf senescence and induces salt tolerance in rice by inactivating JA [30]. *Os12g0569700*, also known as *OsHsp17*.*0*, may enhance tolerance to salt and drought stresses via production of higher proline and lower malondialdehyde levels in rice [31, 32]. *Os02g0466400* encodes inositol 1,3,4-trisphosphate 5/6-kinase and is essential for drought and salt stress responses in rice [33]. For the common upregulated genes in roots of both rice genotypes, there were 97 genes with expression levels higher in Dongdao-4 than in Jigeng-88. Among these genes, *Os03g0230300*, encoding OsSRO1c, confers greater tolerance to drought stress in overexpressing lines through interaction with SNAC1 to promote stomatal closure and reduce water loss [34–36].

Among the downregulated genes in both genotypes, 121 and 83 genes showed greater levels of inhibition in the shoots and roots of Dongdao-4, respectively, than in Jigeng-88 under saline-alkaline treatment. Among these genes, several genes were involved in the response to osmotic stress and phytohormone inactivation. *Os09g0457100* encodes the ABA 8-hydroxylase gene *OsABA8ox3*, and compared to the wild type, *OsABA8ox3* RNAi transgenic rice seedlings demonstrated tolerance to abiotic stress via control of ABA concentrations [37]. *Os01g0182600* encodes the OsGIGANTEA (OsGI) protein, which negatively regulates osmotic stress in rice [38]. The study revealed that mutation of *OsGI* led to tolerance to osmotic stress due to increased proline and sucrose contents and accelerated stomatal movement [36]. These results indicated that decreases in the transcript levels of genes involved in the responses to osmotic stress and phytohormone inactivation were mechanisms that reduced the effects of the stress, thus contributing to the greater tolerance of Dongdao-4 plants.

Among the upregulated genes in shoots of both rice genotypes, a total of 88 genes were assigned to metabolic pathways. Among these genes, *Os07g0154100* and *Os08g0445700*, have been reported to be involved in responses to abiotic stress [39–42], with the former encoding 9-cis-epoxycarotenoid dioxygenase, which is a key gene in gibberellic acid biosynthesis, and the latter encoding a stress responsive gene, trehalose-6-phosphate synthase. Expression of *OsNCED4* in Arabidopsis increased ABA levels and enhanced tolerance to drought and moderate salt stress [39], and transgenic rice overexpressing *OsTPSs* accumulated trehalose, which could confer greater tolerance to cold, high salinity, and drought stress [40–42]. For the common upregulated genes in roots of both rice genotypes, genes were significantly enriched in metabolic pathways and betalain biosynthesis. However, these genes may be indirectly involved in the response to abiotic stress. *Os08g0140300* and *Os08g0140500* were assigned to betalain biosynthesis and are involved in serotonin biosynthesis, which plays an important role in adaptation to environmental changes [43, 44]. Indeed, the overexpression of *OsTDC* in rice plants led to accumulation of higher levels of serotonin and delayed senescence in leaves relative to wild-type plants [43, 44]. These results indicated that genes assigned to betalain biosynthesis may facilitate tolerance to saline-alkaline stress.

## Supporting information

S1 File (XLSX)
